# GABA induces a hormonal counter-regulatory response in subjects with long-standing type 1 diabetes

**DOI:** 10.1136/bmjdrc-2021-002442

**Published:** 2021-10-11

**Authors:** Daniel Espes, Hanna Liljebäck, Henrik Hill, Andris Elksnis, José Caballero-Corbalan, Bryndis Birnir, Per-Ola Carlsson

**Affiliations:** 1 Department of Medical Cell Biology, Science for Life Laboratory, Uppsala University, Uppsala, Sweden; 2 Department of Medical Sciences, Science for Life Laboratory, Uppsala University, Uppsala, Sweden; 3 Department of Medical Cell Biology, Uppsala University, Uppsala, Sweden; 4 Department of Medical Sciences, Uppsala University, Uppsala, Sweden; 5 Department of Women’s and Children’s Health, Uppsala University, Uppsala, Sweden

**Keywords:** diabetes mellitus, type 1, hypoglycemia, receptors, GABA-A

## Abstract

**Introduction:**

Experimentally, gamma-aminobutyric acid (GABA) has been found to exert immune-modulatory effects and induce beta-cell regeneration, which make it a highly interesting substance candidate for the treatment of type 1 diabetes (T1D). In many countries, including those in the European Union, GABA is considered a pharmaceutical drug. We have therefore conducted a safety and dose escalation trial with the first controlled-release formulation of GABA, Remygen (Diamyd Medical).

**Research design and methods:**

Six adult male subjects with long-standing T1D (age 24.8±1.5 years, disease duration 14.7±2.2 years) were enrolled in an 11-day dose escalation trial with a controlled-release formulation of GABA, Remygen. Pharmacokinetics, glucose control and hormonal counter-regulatory response during hypoglycemic clamps were evaluated at every dose increase (200 mg, 600 mg and 1200 mg).

**Results:**

During the trial there were no serious and only a few, transient, adverse events reported. Without treatment, the counter-regulatory hormone response to hypoglycemia was severely blunted. Intake of 600 mg GABA more than doubled the glucagon, epinephrine, growth hormone and cortisol responses to hypoglycemia.

**Conclusions:**

We find that the GABA treatment was well tolerated and established a counter-regulatory response to hypoglycemia in long-standing T1D. Further studies regarding not only the clinical potential of Remygen for beta-cell regeneration but also its potential use as hypoglycemic prophylaxis are warranted.

**Trail registration number:**

NCT03635437 and EudraCT2018-001115-73.

**Significance of this study**

**What is already known about this subject?**

- Gamma-aminobutyric acid (GABA) has, in experimental studies, been found to exert immune-modulatory effects and induce beta-cell regeneration, which make it a highly interesting substance candidate for the treatment of type 1 diabetes (T1D).
- Activation of the GABA-A receptor by benzodiazepines has been found to blunt the counter-regulatory response to hypoglycemia.
- In many countries, including those in the European Union, GABA is considered a pharmaceutical drug.

**What are the new findings?**

- GABA treatment is well tolerated in subjects with T1D.
- GABA can paradoxically establish a counter-regulatory response to hypoglycemia in long-standing T1D.

**How might these results change the focus of research or clinical practice?**

- GABA has a potential use as hypoglycemic prophylaxis in clinical practice in addition to its potential beta-cell regenerative effects.

## Introduction

Gamma-aminobutyric acid (GABA) is a mediator in the central nervous system (CNS) and in peripheral tissues with effects exerted through ionotropic GABA-A and GABA-C, as well as metabotropic GABA-B receptors.^1^ GABA is synthesized from glutamate by glutamic acid decarboxylase, a well-known autoantigen in type 1 diabetes (T1D).^2^ In the adult brain, GABA is the main inhibitory neurotransmitter, while its peripheral effects vary depending on the tissue. Outside the CNS, GABA is found at the highest concentrations in beta-cells and immune cells.^3 4^ Most immune cells express GABA receptor subunits and the metabolic machinery required for GABA metabolism. GABA exerts a predominantly inhibitory effect on immune cells by inhibiting the production of inflammatory cytokines.^5 6^ GABA receptor activation also enhances regulatory T cells, contributing to its immunosuppressive functions.^7^ In addition to beta-cells, GABA-A receptors are also expressed in a number of peripheral tissues, including the pituitary, chromaffin cells in the adrenal medulla and hepatocytes.^8–10^ In experimental studies with rodent and human beta-cells, GABA has been shown to reverse diabetes by stimulating beta-cell regeneration, both through beta-cell proliferation and, although more controversial, transdifferentiation.^11–13^ The combined immune-modulatory and beta-cell effects make GABA a highly interesting candidate for the treatment of T1D. However, in many countries, including those in the European Union, GABA is considered a pharmaceutical drug. In this initial safety and dose escalation study, we have evaluated short-term GABA treatment in subjects with T1D as a first step toward testing the potential of GABA as a beta-cell regenerative treatment.

## Methods

### Ethics and study design

GABA is considered a pharmaceutical drug within the European Union. All participants were provided oral and written information and signed a written consent prior to inclusion in the study.

The study was an open-label, investigator-driven phase I clinical trial, primarily assessing the safety of a controlled-release formulation of GABA, Remygen (Diamyd Medical, Stockholm, Sweden), in adult male subjects with long-standing T1D. GABA was dosed in three steps: low (200 mg), medium (600 mg) and high (1200 mg) dose, with subsequent pharmacokinetic evaluation. Remygen was administered orally as a single daily dose to be taken under fasting conditions in the morning and each dose was administered for three consecutive days. The study period consisted of visits at the hospital for 11 consecutive days. Sample size was decided after dialogue with the Swedish Medical Products Agency. The study included two mixed meal tolerance tests (MMTT), four hypoglycemic clamps and three pharmacokinetic evaluations. All study visits were conducted at Uppsala University Hospital. A detailed study design and all inclusion/exclusion criteria can be found at ClinicalTrials.gov.

### Safety monitoring

All subjects underwent general and neurological examinations, as well as blood sampling for laboratory testing, at multiple time points during the safety trial. The study was monitored by Uppsala Clinical Research Center. An external Data Safety Monitoring Board reviewed the data.

### Laboratory testing

Laboratory tests included metabolic parameters (glucose, hemoglobin A1c (HbA1c) and C peptide), hematology status, renal function and markers of liver injury (aspartate transaminase (AST), alanine aminotransferase (ALT), alkaline phosphate, bilirubin). During the hypoglycemic clamp, the counter-regulatory hormones glucagon, growth hormone (GH), cortisol, epinephrine and norepinephrine were analyzed. Routine blood samples were analyzed at the central laboratory of Uppsala University Hospital. Epinephrine and norepinephrine concentrations were analyzed at Karolinska University Hospital laboratory. C peptide concentrations were also analyzed with an ultrasensitive C peptide ELISA (Mercodia, Uppsala, Sweden). Glycemic control was monitored by flash glucose monitoring (FGM) (FreeStyle Libre, Abbott Laboratories, Chicago, Illinois, USA).

### Pharmacokinetics of GABA

GABA concentrations in plasma were determined the first day of every dose increase at 0, 30, 60, 120, 180, 300 min and 24 hours (nadir value) after GABA administration. GABA levels were analyzed by mass spectrometry at the Mass Spectrometry Based Metabolomics Facility at Uppsala University with a method approved by the US Food and Drug Administration.

### Mixed meal tolerance test

An MMTT was performed after overnight fasting at baseline and at day 11, that is, last day of treatment with 1200 mg GABA. C peptide and glucose levels were measured at 0, 15, 30, 60, 90 and 120 min after ingestion of 6 mL/kg (maximum 360 mL) Resource Protein (Nestlé Health Science, Switzerland).

### Hypoglycemic clamp

A hypoglycemic clamp was performed after overnight fasting at baseline and at each GABA dosage step. An individualized insulin infusion dose was calculated based on body weight (2 mIE/kg/min) and a separate glucose infusion was titrated to achieve and maintain euglycemia (5.5 mmol/L) for 30 min, followed by hypoglycemia (2.5 mmol/L) for 30 min. Blood samples were collected at the end of each plateau. GABA was administrated 30 min before start.

### Endpoints

The primary outcomes of the trial were (1) number of serious and adverse events (SAE/AE) and (2) changes in laboratory parameters, physical examinations and vital signs. The secondary outcomes were (1) C peptide response during MMTT determined as area under the curve (AUC), mean and peak values; and (2) hormonal counter-regulatory response. Additional variables such as fasting plasma C peptide, HbA1c, autoantibodies and number of self-reported hypoglycemic events were included in the general assessment.

### Statistical analysis

Safety laboratory tests were compared with baseline with repeated measurements one-way analysis of variance (ANOVA) with Dunnett’s post-hoc test. For FGM data a non-parametric one-way ANOVA with Dunn’s multiple comparisons test was applied. Hormonal counter-regulatory response under normoglycemic and hypoglycemic conditions was computed by multiple t-tests and corrected for multiple testing with the Holm-Sidak method. Data are presented as mean±SEM. P values <0.05 were considered statistically significant.

## Results

### Characteristics of the subjects

Ten male subjects with T1D were screened, of whom four were excluded from participation in the study (C peptide >0.12 nmol/L (n=2), elevated AST/ALT (n=1) and difficult venous access (n=1)). Six subjects were included and all completed the 11-day trial. All subjects had long-standing T1D (age 24.8±1.5 years, disease duration 14.7±2.2 years). Full descriptive data are presented in table 1.

**Table 1:** Descriptive data including autoantibodies of study participants at baseline

Parameter	Subjects (n=6)
Male (%)	100
Age (years)	24.8±1.5
Age at onset (years)	10.3±2.2
T1D duration (years)	14.7±2.2
BMI (kg/m^2^, ref: 20–25)	22.6±1.4
Hemoglobin (g/L, ref: 130–170)	142.8±3.1
Creatinine (µmol/L, ref: 60–105)	74.2±2.2
Bilirubin (µmol/L, ref: 5–25)	14.4±2.0
ALT (µkat/L, ref: 0.15–1.1)	0.37±0.08
AST (µkat/L, ref: 0.25–0.75)	0.43±0.09
ALP (µkat/L, ref: 0.6–1.8)	1.4±0.1
HbA1c (mmol/mol, ref: 27–42)	64.8±6.2
Fasting C peptide >0.01 nmol/L (n)	1
GAD positive (n)	3
IA2 positive (n)	4

Reference values are presented within brackets.

The cut-off for positive GAD autoantibodies was set to >5 IE/mL and for IA2 to >7.5 kE/L according to clinical routine.

ALP, alkaline phosphate; ALT, alanine aminotransferase; AST, aspartate transaminase; BMI, body mass index; GAD, glutamic acid decarboxylase; HbA1c, hemoglobin A1c; IA2, Islet Antigen 2; ref, reference; T1D, type 1 diabetes.

### Safety evaluation

None of the patients had SAE and there were no AEs reported during treatment with the low dose, while four patients reported mild and transient AEs (mostly neurological sensations) at the medium and high dose of GABA (online supplemental table 1). Hemoglobin levels decreased when compared with baseline during the medium-dose and high-dose treatment, which was deemed to be related to the extensive blood sampling during the study (baseline 142.8±3.1 g/L, low-dose 137.5±2.6 g/L, medium-dose 131.3±2.3 and high-dose 124.8±2.5, p<0.001). Blood platelet and leukocyte counts were unaffected by GABA treatment. Also, ALT, AST, bilirubin and creatinine levels were unaffected, whereas alkaline phosphate levels slightly decreased during the high-dose treatment (data not shown). As expected, the GABA pharmacokinetics displayed increasing AUC levels at higher doses (online supplemental table 2).

10.1136/bmjdrc-2021-002442.supp1 Supplementary data

10.1136/bmjdrc-2021-002442.supp2 Supplementary data

### Metabolic parameters

**Figure 1:**
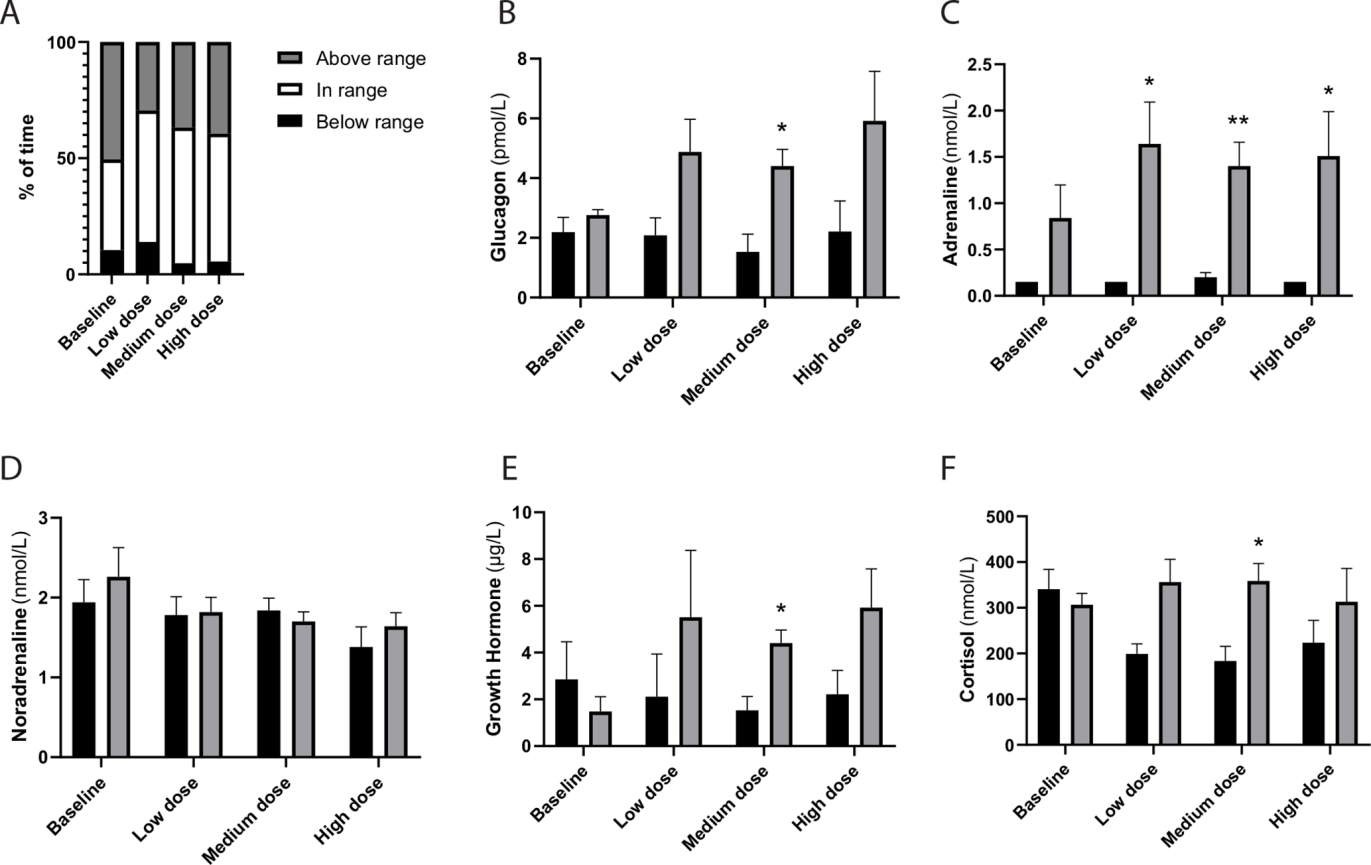
GABA treatment improves the counter-regulatory hormone response to hypoglycemia in type 1 diabetes. (A) Flash glucose monitoring for all subjects (n=6) at baseline and during short-term oral treatment with GABA in increasing doses. (B–F) Hormonal counter-regulatory response during hyperinsulinemic hypoglycemic clamps. Glucose levels were regulated by an intravenous continuous rate of insulin and a variable rate of glucose in order to reach first a 30 min plateau with glucose levels of 5.5 mmol/L, followed by a 30 min plateau at 2.5 mmol/L. Blood samples were collected at the end of each plateau. The analysis contains data from the five subjects without detectable C peptide (<0.01 nmol/L) in which the counter-regulatory response was blunted at baseline. Black bars represent normoglycemia (5.5 mmol/L) and gray bars hypoglycemic conditions (2.5 mmol/L). Data are presented as mean±SEM. *P<0.05 and **P<0.01 compared with the normoglycemic levels at the respective occasion. GABA, gamma-aminobutyric acid.

Five of the patients had undetectable C peptide levels when analyzed with the standard clinical assay (ie, <0.01 nmol/L), and both the fasting and stimulated levels were unaffected by short-term GABA treatment. When assessed by the ultrasensitive C peptide ELISA, three patients were found to have detectable C peptide under fasting conditions and one additional patient during stimulated conditions. The levels were, however, unaffected by the short-term GABA treatment (data not shown). Given the small sample size and limited study period, we could not detect any significant differences with regard to time in range, or time below range, when analyzing the FGM data (figure 1A).

### Response to hypoglycemia

The patient with remaining C peptide displayed a normal hormonal counter-regulatory response to hypoglycemia at baseline, which was maintained under the GABA treatment (data not shown). In contrast, the five patients without detectable C peptide (<0.01 nmol/L) displayed absence of counter-regulatory response at baseline (figure 1B–F). During GABA treatment (600 mg) a counter-regulatory response of glucagon, epinephrine, GH, as well as cortisol occurred in response to hypoglycemia (figure 1B–F).

## Discussion

Short-term oral GABA treatment was not associated with any SAEs. AEs were registered in four subjects, but they were all mild and transient, mainly neurological sensations. In previous studies, fatigue and mild weakness have also been reported.^14^ In another study, 3 g of GABA caused mild transaminase increases in 2% of study subjects.^15^ Glucose control was maintained during the study period and the frequency of hypoglycemia did not increase.

In contrast to previous studies in which activation of the GABA-A receptor by benzodiazepines has been found to blunt the counter-regulatory response,^16^ we paradoxically found that the hormonal counter-regulatory response to hypoglycemia was enhanced by GABA treatment. A tentative explanation for this difference in results is that GABA per se has limited capacity to cross the blood–brain barrier, while benzodiazepines easily cross to exert their effects in the CNS. The mechanism for the counter-regulatory failure induced by benzodiazepines has previously been shown to be lactate release in the ventromedial hypothalamus.^17^ Also, the metabolism of GABA and benzodiazepines differs, which could in turn impact the counter-regulatory response. The present study shows that if the peripheral effects of GABA are isolated, the hormonal counter-regulatory response instead become enhanced by yet unknown mechanisms. A possible explanation could be the activation of GABA-A receptors in other peripheral organs/tissues of importance for the hormonal counter-regulatory response. For instance, GABA-A receptor activation in chromaffin cells within the adrenal glands induces the secretion of catecholamines.^9^ The study period and sample size (number of subjects included) were, however, in this study too small to assess potential clinical benefits in hypoglycemia severity and frequency.

We conclude that the controlled-release formulation of GABA (Remygen) can be considered a safe treatment in T1D and that a larger, long-term clinical trial is warranted in order to further study the clinical potential of GABA as a drug for beta-cell regeneration and, in view of the findings in this study, hypoglycemia protection.

## Data Availability

Data are available upon reasonable request. The data sets generated during the current study can be made available from the corresponding author upon reasonable request.
