# Supplementary material for: GABA induces a hormonal counter-regulatory response in subjects with long-standing type 1 diabetes
Source: BMJ Open Diabetes Res Care. 2021 Oct 11;9(1):e002442. doi: 10.1136/bmjdrc-2021-002442 (PMC8506884; doi:10.1136/bmjdrc-2021-002442)
Supplement: Supplementary data [file bmjdrc-2021-002442supp001.pdf]

Supplementary Table 1

During treatment with the medium- and high dose (600 mg and 1,200 mg) four out of six subjects reported mild and transient adverse events (AEs). In the table all reported AEs are listed and classified. None of the six subjects in the trial had a serious adverse event and there were no AEs during treatment with the low dose (200 mg).

**Subject No.**	**Causal relationship to study drug**	**Maximum AE intensity**	**Description**
1	Possibly related	Mild	Dizziness
1	Probably related	Mild	Flushing
1	Possibly related	Mild	Flatulence
1	Probably related	Mild	Flushing
1	Probably related	Mild	Flushing
1	Possibly related	Mild	Dizziness
4	Probably related	Mild	Flushing
4	Possibly related	Mild	Palpitations
4	Probably related	Mild	Flushing
4	Probably related	Mild	Hypoesthesia
4	Probably related	Mild	Hypoesthesia
4	Probably related	Mild	Hypoesthesia
4	Probably related	Mild	Flushing
4	Probably related	Mild	Palpitations
4	Probably related	Mild	Paraesthesia
5	Unlikely related	Mild	Nasopharyngitis
5	Unlikely related	Mild	Epistaxis
10	Possibly related	Mild	Presyncope
10	Possibly related	Mild	Fatigue
10	Possibly related	Mild	Fatigue
10	Possibly related	Mild	Fatigue
10	Possibly related	Mild	Fatigue
10	Possibly related	Mild	Fatigue
