# Supplementary material for: GABA induces a hormonal counter-regulatory response in subjects with long-standing type 1 diabetes
Source: BMJ Open Diabetes Res Care. 2021 Oct 11;9(1):e002442. doi: 10.1136/bmjdrc-2021-002442 (PMC8506884; doi:10.1136/bmjdrc-2021-002442)
Supplement: Supplementary data [file bmjdrc-2021-002442supp002.pdf]

Supplementary Table 2

GABA pharmacokinetic parameters in six male subjects with long-standing type 1 diabetes during an 11-day dose-escalation trial (200 mg, 600 mg and 1,200 mg) with Remygen®, a controlled-release formulation of GABA. GABA concentrations in plasma were determined the first day of every dose increase at 0, 30, 60, 120, 180, 300 minutes and 24 hours after GABA administration.

Day in study	Statistics	AUC (ng/mL*hour)	Cmax (ng/mL)	Weight corrected AUC	Weight corrected Cmax
**Day 3**	n	6	6	6	6
	Mean (SD)	458.9 (223.7)	61.5 (23.9)	6.4 (4.0)	0.8 (0.4)
	Median (Min, Max)	409.6 (227.1, 767.7)	60.5 (26.6, 99.1)	5.3 (2.5, 13.0)	0.7 (0.4, 1.3)
**Day 6**	n	6	6	6	6
	Mean (SD)	618.6 (217.6)	87.5 (27.1)	8.1 (2.7)	1.1 (0.3)
	Median (Min, Max)	531.4 (429.3, 983.8)	85.8 (58.7, 122.5)	7.1 (5.5, 11.9)	1.2 (0.8, 1.5)
**Day 9**	n	6	6	6	6
	Mean (SD)	782.4 (322.5)	100.3 (43.5)	10.3 (4.3)	1.3 (0.6)
	Median (Min, Max)	846.7 (336.3, 1218.1)	107.9 (48.1, 152.9)	11.2 (4.3, 15.8)	1.4 (0.6, 2.1)
